# Current insights into transcriptional role(s) for the nutraceutical *Withania somnifera* in inflammation and aging

**DOI:** 10.3389/fnut.2024.1370951

**Published:** 2024-05-03

**Authors:** Praful Saha, Saiprasad Ajgaonkar, Dishant Maniar, Simran Sahare, Dilip Mehta, Sujit Nair

**Affiliations:** ^1^PhytoVeda Pvt. Ltd., Mumbai, India; ^2^Viridis Biopharma Pvt. Ltd., Mumbai, India

**Keywords:** nutraceuticals, aging, inflammation, *Withania somnifera*, Ashwagandha, RNA, noncoding RNA, transcription

## Abstract

The health-beneficial effects of nutraceuticals in various diseases have received enhanced attention in recent years. Aging is a continuous process wherein physiological activity of an individual declines over time and is characterized by various indefinite hallmarks which contribute toward aging-related comorbidities in an individual which include many neurodegenerative diseases, cardiac problems, diabetes, bone-degeneration, and cancer. Cellular senescence is a homeostatic biological process that has an important function in driving aging. Currently, a growing body of evidence substantiates the connection between epigenetic modifications and the aging process, along with aging-related diseases. These modifications are now being recognized as promising targets for emerging therapeutic interventions. Considering that almost all the biological processes are modulated by RNAs, numerous RNA-binding proteins have been found to be linked to aging and age-related complexities. Currently, studies have shed light on the ability of the nutraceutical *Withania somnifera* (Ashwagandha) to influence RNA expression, stability, and processing, offering insights into its mechanisms of action. By targeting RNA-related pathways, *Withania somnifera* may exhibit promising effects in ameliorating age-associated molecular changes, which include modifications in gene expression and signaling networks. This review summarizes the potential role of *Withania somnifera* as a nutraceutical in modulating RNA-level changes associated with aging, encompassing both *in vitro* and *in vivo* studies. Taken together, the putative role(s) of *Withania* in modulation of key RNAs will provide insights into understanding the aging process and facilitate the development of various preventive and therapeutic strategies employing nutraceuticals for healthy aging.

## 1 Introduction

### 1.1 Aging

Aging is considered as a progressive deterioration of physiological activity and is characterized by various indefinite hallmarks at both cellular and molecular levels (1). Apart from genomic instability and telomere attrition, ongoing research in this arena reveals various other contributing factors making this process multifaceted and complicated. Senescence occurring at both cellular and organ level leads to age-related diseases like cardiovascular conditions, Alzheimer’s disease, cancer, and sarcopenia, often presenting as comorbidities (2). With the world’s population aged 60 and above projected to double and reach 22% by the year 2050, there is a noticeable increase in both illness and mortality rates among the population (3). Escalating global aging poses a healthcare challenge due to the significant health risks accompanying aging. Therefore, it is necessary to understand the specific mechanisms which drive aging as well as aging-related complications (4, 5). Aging and aging-related complexities are consequences of external factors (environmental) and internal factors (modulation of gene expression) (6). Genetic alteration is considered as crucial in all organic processes starting with maturation of embryo to biological aging (7). Transcriptomic studies including alterations in all RNA species (including mRNA, rRNA, tRNA and non-coding RNAs) inferred that genetic alterations may often lead to aging-related comorbidities namely diabetes, neurological disorders, obesity, cancer and viral infections (6). Although certain illnesses or ailments might exhibit recognizable genetic or molecular markers such as transcriptomic modifications contributing to their simultaneous occurrence, numerous comorbidities stem from complex interplays among genetic, environmental, and lifestyle elements, sometimes without apparent transcriptomic changes (8).

### 1.2 Withania somnifera

**Figure 1:**
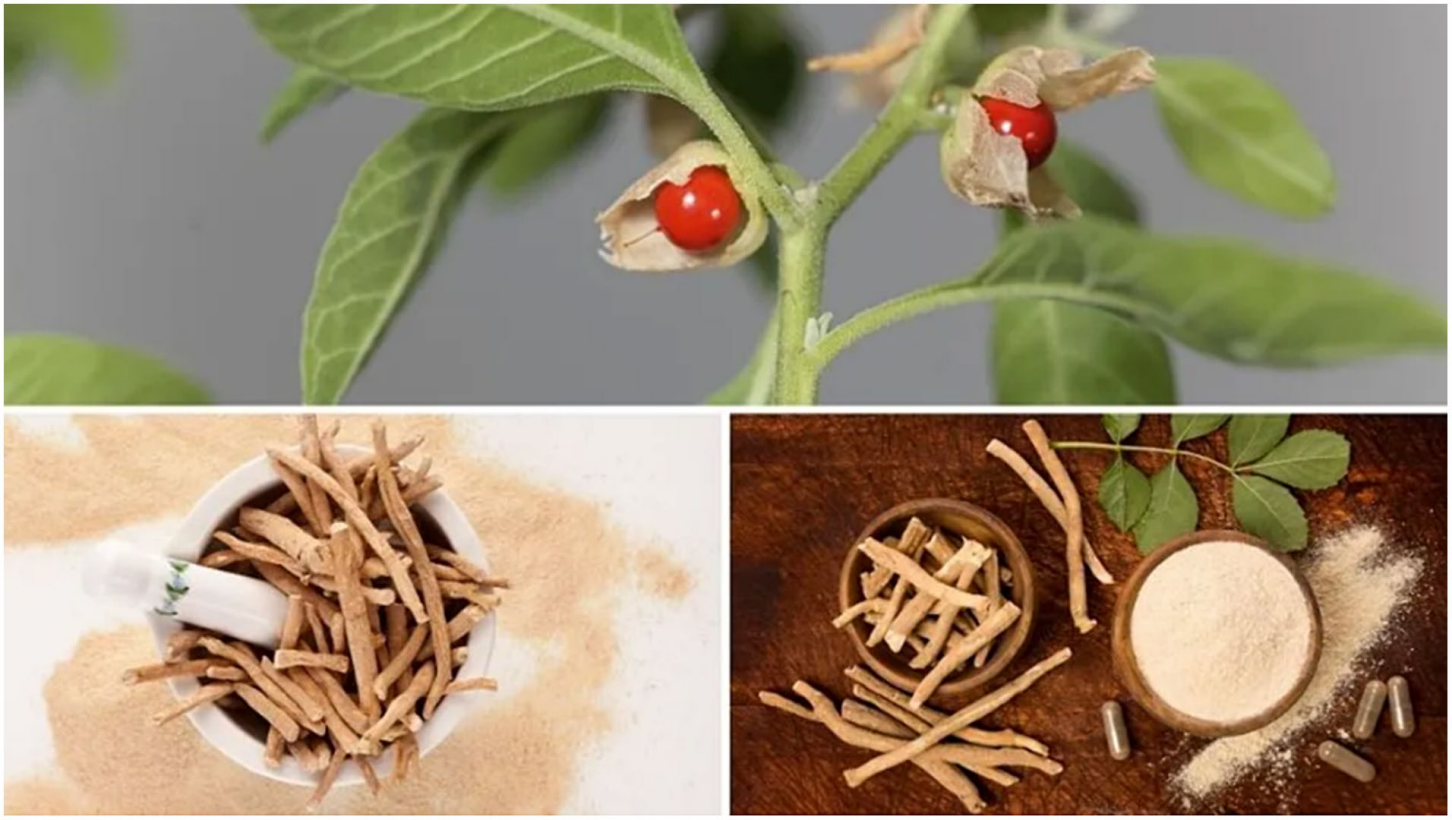
*Withania somnifera* (9).

*Withania somnifera* (WS) (Figure 1) is a perennial, woody shrub that typically reaches a height ranging from 0.5 to 2.0 meters, belonging to family *Solanaceae* and is recognized by names like “Winter cherry” or “Indian Ginseng” (in English); “Ashwagandha” (in Sanskrit) and “Asgandh” (in Hindi) (10). WS is established in the regions of Middle East, Africa, Sri Lanka, China, India, Canary Islands and in the warm areas of Europe and Australia where the climate is hot and dry (11).

**Figure 2:**
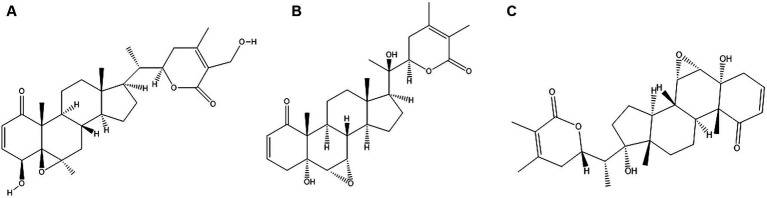
Chemical structures of major constituents of *Withania somnifera* **(A)** Withaferin A; **(B)** Withanlide; **(C)** Withanone. Structures of the constituents were drawn using Chemdraw Version 20.1.1.125.

The active biochemical components found in WS comprise of alkaloids such as isopelletierine, anaferine, cuseohygrine, anahygrine, etc., and steroidal lactones which include withanolides, withanosides (glucose conjugated), withaferin and saponins (12). The chemical structures of major withanolides which include Withaferin A, Withanolide and Withanone are illustrated in Figures 2A–C respectively. Structures of the remaining constituents has been published elsewhere (11). The anti-stress agents in WS comprise sitoindosides and acylsterylglucosides and withaferin A (WA). It was reported that using WA and sitoindosides can alleviate stress in experimental models (13).

### 1.3 Mechanism for pharmacologic activity of *Withania*

#### 1.3.1 Immune regulation by *Withania*

The immune system is an important factor in the origin and mechanisms of different diseases, contributing significantly to biomedical research advancement (14). Conditions affecting the skin, gastrointestinal tract, respiratory system, joints, vital organs, and infectious illnesses are increasingly recognized as primarily influenced by immunological factors (15). A wide range of studies have shown that most of the major components of WS have immunomodulatory effects in mice (16) and humans (17). The immunomodulatory role of WS in various inflammatory complications such as inflammation (reducing type 2 cytokines, and inflammatory markers such as TNF-α and IgE) has been reported (18). The extract derived from WS root showcases robust hemopoietic, antioxidant, adaptogenic, and immune-stimulating properties (19). WS plays a role in immunomodulation by significantly improving the immune profile by altering innate and adaptive immune systems (20). In a randomized and controlled clinical trial, the immune-stimulatory effect of WS was evaluated and it was reported that WS enhanced CD4+/CD8+ and expression of CD3+/CD19+/CD45+, thus, increasing the antibody and antibody-forming cells (20). The extract from WS has demonstrated a notable ability to boost cell-mediated immunity in mice (21). Levels of interferon gamma (IFN-y), interleukin-2 (IL-2), and granulocyte macrophage colony-stimulating factor (GM-CSF) have been elevated in experimental mouse models when treated with WS; this indicates potential immune-enhancing and myelo-protective effects of root extracts of WS (22). Furthermore, it enhances the body’s immune system, specifically enhancing cell-mediated immunity, which is vital in defending against diseases (23).

#### 1.3.2 Modulation of nitric oxide (NO) signaling by *Withania*

WS amplifies the nitric oxide synthetase (NOS) activity in macrophages, consequently augmenting the microbial eradication capability of these immune cells. It has been elucidated that WS enhances the activity of nitric oxide via NOS induction (22). Indeed, WS boasts a wide array of beneficial effects on the body. Additionally, it promotes a balance in reproductive and sexual function contributing to an improved reproductive health (23). WS’s adaptogenic properties make the body more resilient to the harmful effects of stress (24). Furthermore, viscosalactone B and 27-O-glucoside (a derivative of WA) extracted from alcoholic leaf extract have demonstrated significant antiproliferative activity when treated with different cell lines viz. CNS cancer SF-268, breast cancer MCF-7, colon cancer HCT-116 and lung cancer NCI-H460 cell line (11).

#### 1.3.3 Antioxidant activities of *Withania*

Studies have shown antioxidant activities of WS concluding that it helps in prevention of glycation-induced pathogenesis as well as decreasing the levels of many oxidative stress markers indicating its potential therapeutic effects in managing healthy aging (25–27). WS’s potent antioxidant properties have an important part in protecting cells against free radicals (23). Hydroalcoholic root extracts of WS contains a diverse range of molecules namely saponins, particularly sitoinsides 7 and 8, withanolides and alkaloids which have anti-stress, anti-inflammatory and anti-oxidant properties, respectively, (28). Anti-inflammatory potential of withanolides and alkaloids is reported in *in vitro* study using RAW 264.7 cell line. Anti-stress properties of sitoindosides 7 and 8 were demonstrated using Wistar albino rats and albino mice (28). Also, studies conducted to investigate the effect of alkaloids in RAW 264.7 cells showed that the expression of iNOS and COX-2 proteins were upregulated (29). In addition, alkaloids inhibited LPS-induced inflammation, indicating a putative anti-inflammatory potential of alkaloids (29). Alkaloids from WS (withanamides A-I) are also reported to lower the rate of lipid peroxidation along with possessing anti-oxidant properties (30).

WS is known for its versatility in treating a range of conditions, such as immunomodulation, rejuvenation, enhancement of cognitive function, inflammation, enhancing concentration, etc. However, a synthetic review exploring its potential role in ameliorating aging and aging-related disorders is currently lacking. In this review, we summarize mechanistic pathways modulated by WS via coding and non-coding RNAs in aging relying on information gleaned from both *in vitro* and *in vivo* studies. The RNA biomarkers regulated by WS can potentially serve as promising targets to mitigate aging-related degenerative changes and, perhaps, to some extent, reverse biological aging. This may facilitate the development of various preventive and therapeutic strategies employing WS as a nutraceutical for healthy aging.

## 2 Aging

Aging can be described as a gradual decline over time in physiological functions essential for survival and reproductive capabilities (31). The attributes associated with aging, distinct from age-related illness like cancer and heart diseases, impact every species (31). In mammals, the occurrence of aging is heterogenous wherein it is accompanied by organs and tissues deterioration and their functional capabilities gradually decrease over time but not necessarily degrading into pathophysiology in healthy aging (32). Consequently, aging is considered as an important factor in pathogenesis of many diseases such as dementia, osteoarthritis, cardiovascular disease, cancer, type 2 diabetes, idiopathic pulmonary fibrosis and glaucoma (33). To classify aging through a biological measurement, a biomarker needs to possess specificity, systemic relevance, and practical utility (34). Biomarkers of cellular aging have been categorized into ten different aspects *viz.* telomere shortening, genetic instability, nuclear body disorders, cell cycle arrest, epigenetic changes, mitochondrial impairment, signaling pathway rerouting, epigenetic changes, loss of proteostasis, metabolic alterations and senescence-associated secondary phenotype (35, 36).

### 2.1 Hallmarks of aging

**Figure 3:**
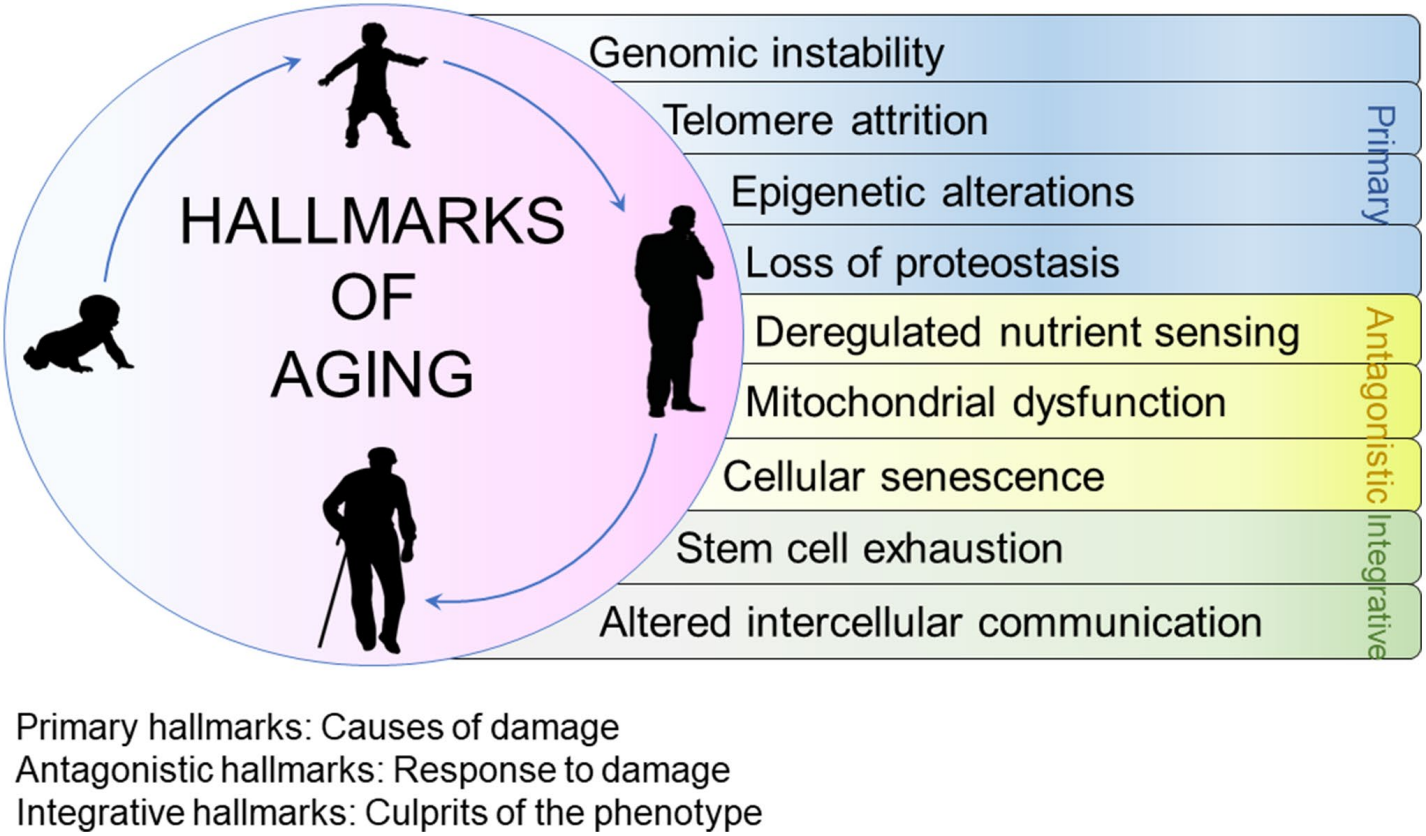
Hallmarks of aging.

Exploring biological aging and understanding the factors related to decelerating organism function and the development of diseases is essential (37). Scientists have made significant progress in unravelling the solutions through elucidation of nine different processes that contribute to the gradual decline and loss of bodily functions. These functions are also known as hallmarks of aging (38). The hallmarks are categorized into 3 groups (38). The first category includes primary hallmarks (causes of age-associated damage); second, antagonistic hallmarks (response to damage) and third, integrative hallmarks (response consequences and culprits of aging). Primary hallmarks include genomic uncertainty, attenuation in telomere strength, epigenetic alterations and proteostasis deprivation. Antagonistic hallmarks include deregulated nutrient sensing, affliction in mitochondrial function and senescence. Integrative hallmarks include stem cell exhaustion and alter intercellular communication (33). All the above-mentioned hallmarks of aging are illustrated in Figure 3.

#### 2.1.1 Primary hallmarks

The primary causes of aging are related to various degradations, alterations and instabilities to our genomic framework and further cascading processes (39). Genomic instability and telomere shortening are major causes of DNA damage, while epigenetic alterations cause post-translational modification of histones and chromatin remodeling (40). Proteostasis irregulation can also have major impacts on aging due to refolding of proteins (41). Telomeres, protein structures which protect the terminal regions of the DNA, go through an attrition process as a part of cell cycle, while oxidative stress, inflammation and chronic stress are also known to increase the rate of attrition leading to degradation in the protective regions making DNA more susceptible to damage (42, 43). To add to it, genomic instability caused by both internal and external factors can alter the genetic code; this kind of DNA damage hinders the process of protein production used to damage repair and accelerates the process of aging (40, 44). Structural changes due to epigenetic alternations degrade the cellular function and are associated with a range of age-related diseases such as cancer, diabetes, osteoporosis, neurological diseases, and increased inflammation (45). Proteostasis, also known as protein homeostasis, helps maintain protein integrity to ensure proper functioning (46). Dysregulation due to various internal and external factors leads to refolding which can have serious health implications such as neurodegenerative disorders, impaired muscle function and low cardiac health (41).

#### 2.1.2 Antagonistic hallmarks

Response of the cells to nutrients is considered as nutrient sensing (47). Mammalian target of Rapamycin (mTOR) is a crucial unit which incorporates nutrient sensing with different cellular processes that can lead to growth and proliferation of cells (48). In growing organisms, cell proliferation and cell growth are very crucial and, therefore mTOR is activated (49). mTOR is naturally decreased in aging (50). Over activating this pathway can cause increased aging and increase in cancer rate, heart conditions, metabolic disorders, bone loss, neurodegenerative conditions and sarcopenia (50). The mitochondrion serves as the cell’s energy hub, where most of the body’s energy is generated (51). Aging tends to decrease the efficiency of the mitochondria due to reduced biogenesis (51). Accumulation of reactive oxygen species and deficiencies (DNA polymerase γ) are the major causes of mitochondrial dysfunctions (52). Cellular senescence, or cell aging, is a process wherein the cell cycle is arrested and accompanied by characteristic phenotypic changes (53). The number of senescent cells tends to rise with age, leading to an increase in inflammatory markers that contribute to the aging process (1).

#### 2.1.3 Integrative hallmarks

Adult stem cells are necessary for continuity of tissue homeostasis and regeneration (54). Consequently, the qualitative and quantitative decrease in functions of stem cells during the entirety of life are referred to as its exhaustion (1). Decline in stem cell regeneration is considered as a marker of aging since the proportion of stem cells and their pace of division gradually decreases over time (55). Stem cell exhaustion in adults can lead to cognitive decline, neurological degenerative diseases, delayed healing, lowered immune functions, and unfavorable heart conditions (55). Cells communicate with each other based on different chemical and electrical means such as endocrine, neuroendocrine and neuronal level. Thus, neurohormonal level is deregulated in aging as inflammatory reactions increase (56). One of the most prominent changes in cell signaling biomarkers is “inflammaging” indicating chronic inflammation development in elderly population (57).

### 2.2 Cellular senescence

Of the nine hallmarks of aging, cellular senescence has been implicated as a major cause of aging and age-related diseases (33). The phenomenon of senescence was initially elucidated in 1961 by Hayflick and Moorhead using human diploid fibroblasts cells who reported that the cells undergo at least 40–60 divisions before arresting their cell cycle (58). The process of senescence is characterized by cell-cycle arrest in the G_1_ or probably G_2_ phase, which prevents the proliferation of damaged cells (59). Cellular senescence can also occur at embryonic development and is activated by cellular impairment including the processes of DNA damage response (DDR), telomere attrition or dysfunction, activation of various oncogenes, or loss of ability of tumor suppressor genes, epigenetic changes and organelle damage (60). The major risk for cellular senescence is DNA damage which initiates the canonical p53-p21 pathway and DNA damage response (61). p21 downregulates CDK complex inhibiting the formation of DREAM complex suppressing genes involved in cell cycle through binding with their homology regions (62).

A permanent shunt of cell cycle confirms that cells altered due to genomic changes do not pass on their genomes (63). The process is initiated by the activation of p16^/^Rb and/or p53/p21 pathways (64). An analysis of several genome wide association studies inferred that *INK4a/ARF* locus, also known as genomic locus, contributes to a plethora of aging-related pathologies (65). p15^INK4b^ and p16^INK4a^ inhibit CDK4/6 affecting the functionality of cell cycle. In contrast, activation of ARF leads to inhibition of murine double minute 2 (MDM2) which leads to crosstalk between p53/p21^CIPI^ pathways (66). p16^INK4a^ is accumulated during aging and is recognized as a biomarker in aging and aging-related diseases (33).

### 2.3 Oxidative stress

Oxidative stress occurs when the ROS production and cellular defense mechanisms are imbalanced (67). ROS are essential for maintenance and growth of cells as they play important roles in cellular defense mechanisms (68). Free radical formation is a crucial step for the cell protection and cell survival within its physiological limits (69). This process escalates with age and affects the normal functioning of various tissues in the body (70). Furthermore, various prolonged diseases related to aging such as cardiovascular disease, diabetes, pulmonary, bones and muscle related disorders are linked to oxidative stress (71). Oxidative stress is induced when there is an elevation in ROS production and reduction in ROS neutralization (67). Since the ROS are produced rapidly, their toxic levels are attained and lead to higher Ca^2+^ stimulation in TCA cycle, this leads to the increased activity of ETC and NADPH production (72). Autooxidation with high production of glucose guides to the alteration of metabolic activity that leads to inhibition of enzymes glutathione peroxidase (GPx) and catalase (CAT) while ROS causes DNA damage (73). In aging, there is deposition of ROS-induced damage which leads to age-associated functional losses (74). Aging and associated disorders which are induced by oxidative stress cause degradation in soft tissues and disruption of homeostasis (75). Moreover, oxidative stress activates irregular mitochondrial signaling leading to alteration of homeostasis of mitochondria and causes age-dependent cellular damage (75).

### 2.4 Metabolic stress as a marker in aging

Diseases related to abnormal functions in metabolism have been on the rise because of various lifestyle changes that include caloric surplus diet, smoking, etc. (76). This leads to metabolic symptoms such as obesity, diabetes, insulin resistance and hypertension. These diseases are also known as metabolic syndrome (77). Even after many clinical advancements to improve the conditions for cardiovascular disease, patients who are suffering from metabolic syndrome have low life expectancy as well as suffer from premature aging (78). Metabolic disorders are linked to an early onset of cardiovascular aging risk, which encompasses cardiac remodeling, improper functioning of cardiac pump, endothelial damage, and calcification, consequently elevating the susceptibility to heart failure (79). Alterations in metabolic homeostasis is one of the major cause of premature aging (80). Autophagy is referred to as a “process of degradation of injured cell organelles and proteins” and it lowers with aging. Inhibition of this process leads to shortening of life-expectancy rate and resulting in premature aging (81). The process of autophagy tends to be actively involved in pathogenesis of disease such as cardiac arrest, CHD, atherosclerosis (82). Metabolic stress-induced impairment in the autophagic clearance mechanism has a role in shifting the cellular, tissue, or organ health from a normal state to a pre-senescent condition marked by visible pathology (80). Multiple advantages have been postulated in extending lifespan and mitigating age-related issues, both in clinical practice and experimental research (78). Dysregulation of metabolic and physiological stress have shown to affect telomere length and maintenance which leads to aging (83). Additionally, mTOR signaling is a major regulator of cell metabolism and autophagy by consistently inducing autophagy in cells (84). mTOR signaling is prone to alterations made in nutritive environments such as IGF-1, fatty and amino acids (49). mTOR regulation and aging are very closely related as the level of mTOR elevated in hematopoietic stem cells in elderly population suggesting that modulation in mTOR pathway can serve as a potential therapeutic marker in aging (85).

## 3 Biosynthesis and metabolism of *Withania* compounds

**Figure 4:**
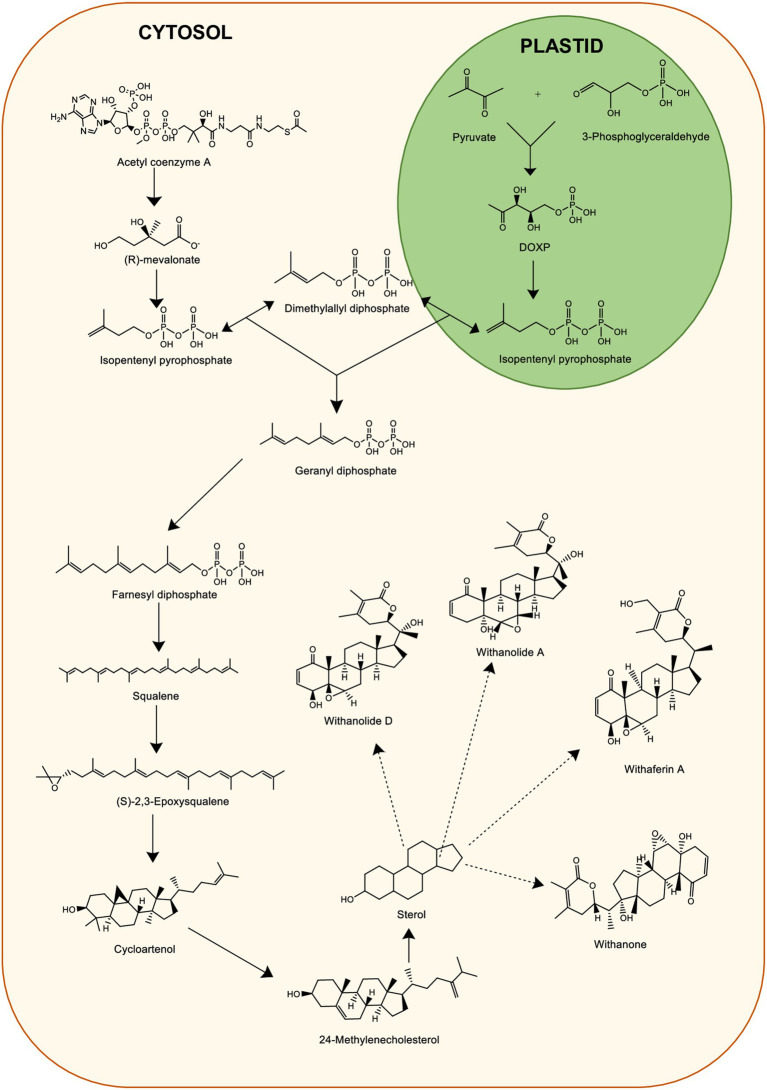
Biosynthesis of Withanolides from plant-derived sources.

There has been extensive use of WS for its several medicinal properties which are responsible for many beneficial pharmacological activities (86). Withanolides are crucial secondary metabolites demonstrating pharmacological activity (87). To date, multiple withanolides, including prominent ones like withanolides, withaferin A (WA) and withanone are extracted from WS (88). Therefore, due to the growing demand of WS in the medicinal industry, it led to the search of a wide range of approaches for the mass production of roots of WS (89). Activation of metabolic pathway leads to the WS constituents in various parts. The major withanolides are produced via mevalonate (MVA) and methylerythritol 4-PO_4_ (MEP) pathways occurring in plastids and cytosol, respectively, (90). Chemically, withanolides are also known as triterpenoids which are 30-carbon compounds (91). Triterpenoids generally are biosynthesized through a metabolic pathway. This require isoprene units such as isopentenyl pyrophosphate (IPP) and dimethyl allyl pyrophosphate (DMAPP) as precursors (91). The MVA pathway engages seven enzymes to create precursor molecules IPP and DMAPP for terpenoid biosynthesis (92). Initially, it starts by condensing two acetyl-CoA molecules into acetoacetyl (AcAc)-CoA, facilitated by the enzyme AcAc-CoA thiolase to form 3-hydroxy-3-methylglytaryl-coenzyme A (HMG-CoA). Further, HMG-CoA reductase catalyzes a double reduction reaction which results in mevalonate biosynthesis from HMG-CoA that converts into IPP. IPP further converts to DMAPP. IPP from MEP pathway is converted to farnesyl pyrophosphate (FPP) (90). Squalene biosynthesis occurs by the FPP condensation in an NADPH-dependent manner. The delta-lactonization of 24-methylenecholesterol synthesis is an important step leading to withanolide biosynthesis (90). The biosynthetic pathway of withanolides including MVA and MEP pathway is illustrated in Figure 4.

Senthil et al. (93) carried out transcriptomic analysis of *WS* leaf and root tissues cultured *in vitro*. This provided insights on gene expression involved in biosynthesis and accumulation of essential withanolides (93). Various critical genes, such as hydroxymethylglutaryl reductase (HMGR), farnesyl pyrophosphate synthase (FPPS), glucosyltransferases (GT), cycloartenol synthase (CAS), squalene (SE) have specific roles in the process (94). For instance, HMGR facilitates HMG-CoA to mevalonate conversion, while FPPS led to the production of FPP, a fundamental element in numerous pathways (95). Phytosterols and triterpenoids are produced with the help of SE and CAS genes. Furthermore, GT facilitates the glycosylation of various natural compounds, aiding in the detoxification process (96). FPPS, another crucial enzyme, starts the biosynthesis of triterpenoid precursors to support withanolide production within the isoprenoid pathway (97). Expression of these genes involved in withanolide production displayed varying patterns along with essential withanolides like withanolide A and withaferin A. CAS, following the production of 2,3-epoxy squalene, induces the following step which leads to 24-methylene cholesterol synthesis, a precursor vital for withanolide synthesis (93). Also, a study (98) was conducted where virus induced gene silencing technique was used to assess the genes involved in withanolides’ biosynthesis. Through quantitative RT-PCR studies, it was revealed that biosynthesis of terpenoids is modulated by complex coordination of various enzymes and flux distribution of several metabolic channels (98).

Soni et al. (99) evaluated the function of endophytic *Bacopa monnieri* after isolation and identification of the fungal endophytes with plant growth promoting potential. By isolating endophytes under *in-vitro* conditions, secondary metabolites were produced. The study inferred that *B. monnieri* helps in withanolide production and biosynthesis in a cost-effective manner (99).

Gupta et al. (100) performed transcriptomic analyses for WS root and leaf to synthesize Withanolide A and Withaferin A, respectively, to further facilitate the understanding of Withanolide synthesis pathway. Analyses of the data shed light on the genes participating in synthesis of withanolide (100). Also, the investigation pinpointed genes such as CYP450, glycosyltransferase (GT), and methyltransferase (MT) that showed distinctive occurrence or expression in roots and leaves, potentially contributing to tissue-specific withanolide synthesis. The resulting sequencing for WS highlighted insights into the biosynthetic pathways of secondary plant products that are specific to tissues. Furthermore, it presents opportunities for devising strategies to enhance withanolide biosynthesis through biotechnological interventions (100).

## 4 Role of *Withania somnifera* in modulating RNAs in aging and inflammation

### 4.1 Non-coding RNAs in aging and inflammation

There is a scarcity of literature on non-coding RNA in aging and inflammation. MicroRNAs (miRNAs) play major role(s) in progression of many medical complications, including osteoarthritis (OA) (101). OA is traditional age-related disorder as the chances of OA increases with age due to sarcopenia and structural loss of bones in increasing age (102). Destruction of articular cartilage, limitation of movement, bone inflammation and synovitis are characteristics of OA (103). miR-25 modulates the expression of inflammatory markers and is downregulated in OA conditions (104). To check the role of Withaferin A in modulation of miR-25, a study was conducted (105) to investigate the role of Withaferin A in osteoarthritis using rabbit articular chondrocytes. It was reported that treatment of Withaferin A increased miR-25 expression by inducing the expression of cyclooxygenase 2 (COX2) providing a therapeutic approach in the treatment of OA (105). Further, miR-181c-5p is associated with the promotion of NF-κB mediated inflammation by downregulating protein tyrosine phosphatase nonreceptor type 4 (PTPN4) and therefore miR-181c-5p level may serve as a potential therapeutic factor in inflammation (106). However, Shuaib et al. elucidated the impact of WS on miR-181c-5p, and it was inferred that Withaferin A potentially upregulated the expression of miR-181c-5p (107).

### 4.2 Messenger RNAs involved in inflammation and aging-associated diseases

#### 4.2.1 mRNAs involved in inflammation

One of the prevalent features related to aging is inflammation and, in the absence of any kind of open infection, this chronic inflammation is referred to as “inflammaging” (108). It poses a major risk factor in major health degradation and mortality in elderly populations (109). Human keratinocytes have a crucial role in inflammation by mediating tumor necrosis factor (TNF)-α and interleukins (IL) (110). Many skin diseases that are related to inflammation such as psoriasis, atopic dermatitis and allergic contact dermatitis are highly associated with keratinocytes and cytokines (111). Therefore, to study the anti-inflammatory effects of WS in modulation of these cytokines, Sikandan et al. (112) demonstrated the effect of Ashwagandha water extract (ASH-WEX) in human keratinocyte (HaCaT) cell line and 6-week-old male C57BL/6 J mice. RNA was isolated after the fifth day of treatment from mice skin and RT-qPCR was performed to check the transcriptional regulation of inflammatory cytokines in both HaCaT cells and C57BL/6 J mice. It was inferred that ASH-WEX inhibited TNF-α, and interleukins (IL-6, -8, -1β, and -12) while the expression of TGF-β1 was reduced in a dose-proportional manner (112). Also, mRNA expression studies performed in mice showed that ASH-WEX treatment inhibited TNF- α expression while it overexpressed TGF-β at mRNA level (112). Furthermore, a study was conducted (113) to demonstrate the beneficial effects of dry leaf extract of WS on anxiety and neuroinflammation which results from obesity. Using mRNA expression analysis by qRT-PCR, it was observed that WS led to inhibition of IKKα/β which further suppressed NF-κB signaling, and reduced the expression of iNOS, GFAP, IL-1β, TNF-α, PPARγ and MCP-1. Also, WS treatment led to upregulation of Bcl-xL and downregulation of Bad mRNA which further inhibited high fat diet-induced apoptosis in rats (113).

**Figure 5:**
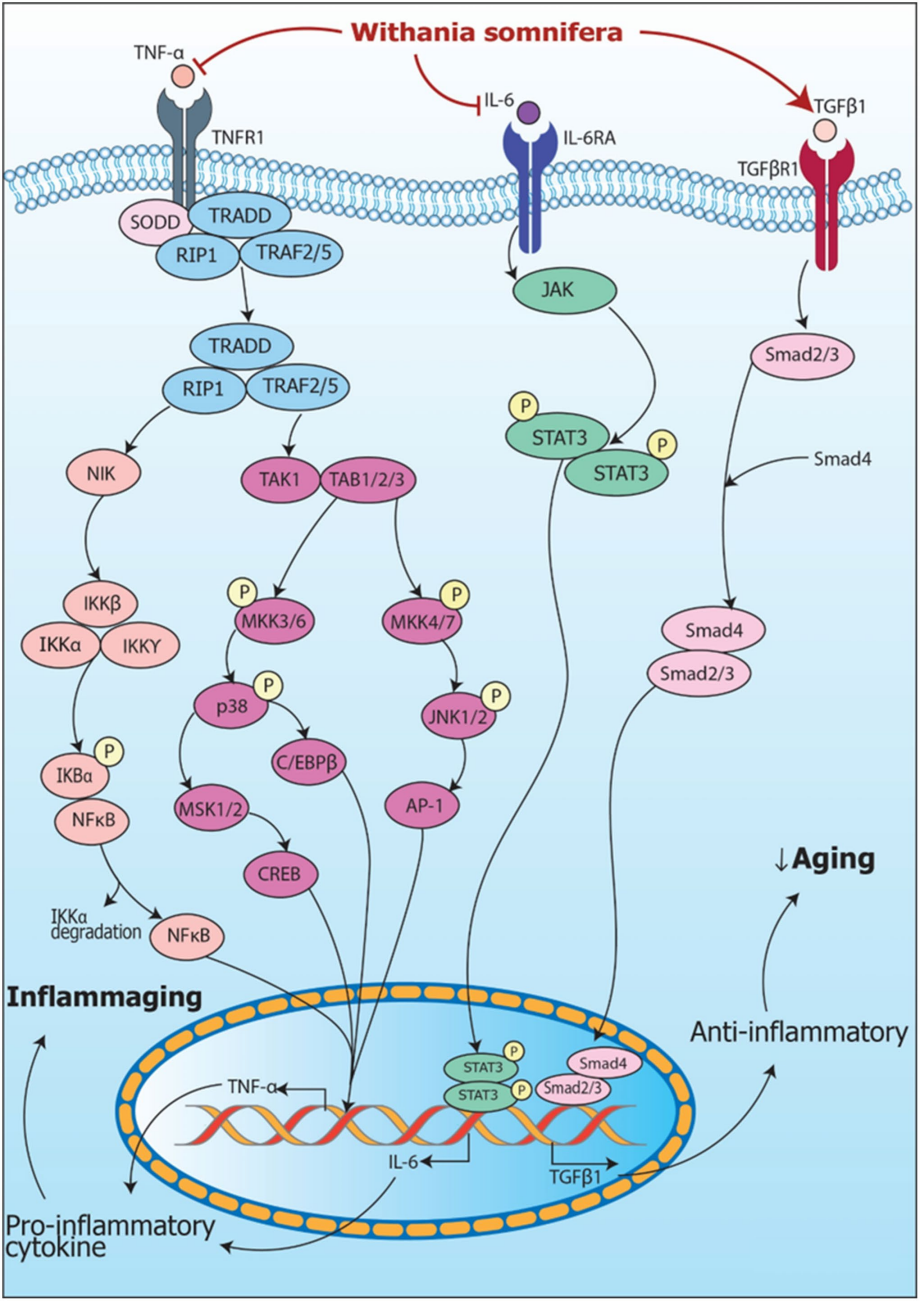
Signaling pathways implicating *Withania somnifera* in modulation of RNAs in inflammation. (1) TNFα, a tumor suppressive cytokine, binds to TNFR1 releasing SODD and activating TRADD complex which further activates NIK and TAK/TAB1/2/3 complex resulting in NF-κB and MAPK signaling pathway activation that transcribes TNFα mRNA via NF-κB, CREB, C/EBPβ and AP-1 signaling. (2) TNFα is inhibited by *Withania somnifera* at transcriptional level which further inhibits inflammation. In addition, IL-6 binds to IL-6R activating JAK/STAT signaling pathway which transcribes IL-6 mRNA. *Withania somnifera* inhibits IL-6 at transcriptional level which, in turn, inhibits JAK/STAT signaling. Moreover, TGFβ1, anti-inflammatory cytokine binds to TGFβR1 initiating SMAD signaling leading to transcription of TGFβ1 mRNA. *Withania somnifera* induces the expression of TGFβ1 resulting in attenuation of inflammation.

Signaling pathways including modulation of RNA by *Withania somnifera* in inflammation are summarized in Figure 5.

#### 4.2.2 mRNAs involved in neurodegeneration

A key neurodegeneration hallmark includes accumulating advanced glycation endproducts (AGE) in the brain (114). Similarly, receptors for advanced glycation endproducts (RAGE) are overexpressed in brain (115). Gathering of amyloid-beta (Aβ) is considered as a major indicator of neurogenerative disease (116). Two crucial proteins, nuclear factor-κB (NF-κB) and (NLRP3), play significant roles in the neurodegenerative disorders development which include Alzheimer’s disease (AD) (117). Additionally, epigenetic modulation which includes histone deacetylase 2 (HDAC2) alteration is considered as a pivotal factor in Alzheimer’s disease (AD) pathogenesis (118). Hence, to investigate the therapeutic role of WS in modulation of these genes in AD, Venkata et al. (119) performed gene expression analysis using SH-SY5Y cells overexpressing amyloid precursor protein (SH-APP) cells. The results revealed that treatment with WA decreased NF-κB and IL-1β expression significantly, concluding that both of these genes have crucial roles in NF-κB-mediated neuroinflammation. Additionally, Withaferin A administration in SH-APP cells led to a downregulation of Aβ, an important mediator in the neuroinflammatory processes implicated in the pathogenesis of AD (119).

The memory process can be affected by a range of pathological factors, such as neurodegenerative disorders, stroke, tumors, head injuries, oxygen deficiency, cardiac surgery, nutritional deficiencies, neurological complexities such as depression and anxiety as well as side-effects of different medications and the natural process of aging (120). Scopolamine, a cholinergic antagonist, induces amnesia in humans as well as in rat models (121). These models have found extensive application in comprehending the molecular, biochemical, and behavioral alterations, serving as valuable tools for identifying potential therapeutic targets in the context of memory impairment (121). To evaluate the role of *Withania somnifera* in memory impairment, Konar et al. (122) assessed the therapeutic targets using alcoholic extracts of *Withania* leaves (i-Extract) in scopolamine-induced male Swiss albino mice of 12 weeks. From RT-PCR analysis, it was reported that scopolamine downregulated the mRNA expression of brain derived neurotropic factor (BDNF) and glial fibrillary acidic protein (GFAP) in mouse cerebrum in proportion to time and dose. Furthermore, it was also observed that i-Extract treatment attenuated the downregulation of scopolamine treatment in proportion to dose suggesting that WS serve as putative therapeutic agents to minimize the progression of neurodegenerative disorders (122).

**Figure 6:**
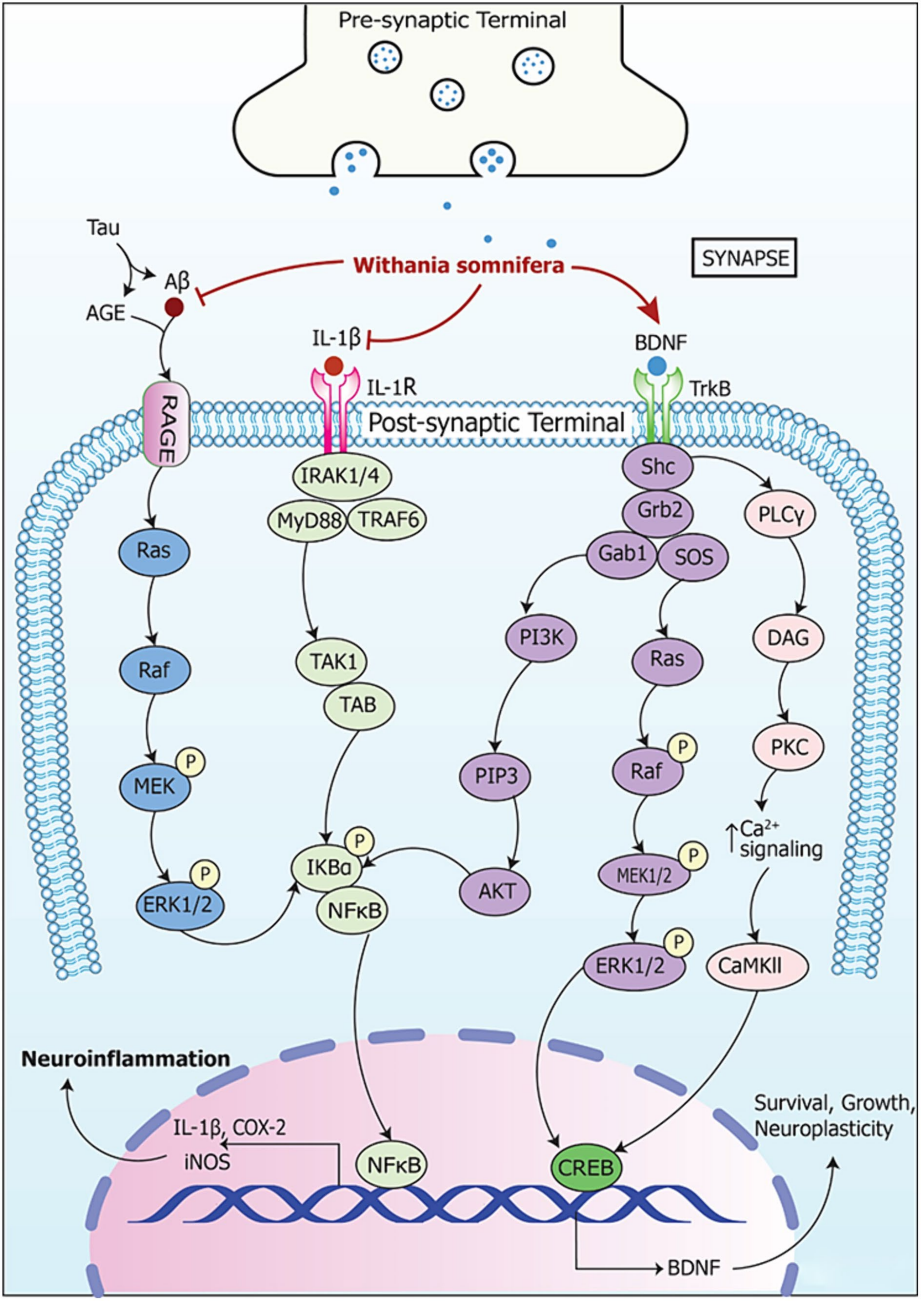
Signaling pathways implicating *Withania somnifera* in modulation of RNAs in neurodegeneration. AGE (advanced glycation endproduct) is made and secreted by microgial cells when activated. This induces RAGE expression in neurons leading to cell death causing neurodegenerative diseases. Also, iNOS is activated by AGE which further enhances apoptosis and degeneration of neuronal cells. AGE, when increased in quantity, augments formation of amyloid-beta (Aβ), tau protein and amyloid precursor protein (APP). This, in turn, induces tau hyperphosphorylation as well as AGE-Aβ cross-linking. Further, Aβ binds to RAGEs to activate ERK1/2 pathway. This leads to phosphorylation of NF-κB and proteasomal degradation of IKBα. This, in turn, enables NF-κB translocation toward nucleus and their gene target is expressed simultaneously. Moreover, IL-1β binds to IL-1R and activates IRAK1/4/MyD88/TRAF6 complex leading to NF-κB signaling via TAK1/TAB. This results in expression of their target genes which contribute to neurodegeneration. *Withania somnifera* inhibits the expression of Aβ and IL-1β at transcriptional level which leads to further inhibition of the downstream pathways. Thus, it reduces the expression of pro-inflammatory genes. Furthermore, *Withania somnifera* induces the activity of brain-derived neurotropic factor (BDNF) which is inhibited in neurodegeneration. The binding of BDNF to tropomyosin-related kinase receptor type B (TrkB) leads to homodimerization and activation of adaptor protein such as Src homology domain 2 (SH2). Thereafter, SH2 activation leads to the activation of phosphoinositide 3-kinases (PI3K)-AKT, Ras-mitogen-activated kinase (Ras-MAPK) and phospholipase Cγ1-protein kinase C (PKC) signaling pathway.

Signaling pathways involving RNA in neurodegeneration and their modulation by *W. somnifera* are summarized in Figure 6.

#### 4.2.3 mRNAs involved in stress

The aging process is linked to alterations in endoplasmic reticulum (ER) chaperones and expression of folding enzymes (123). This leads to proteostasis disruption and elevation of misfolded proteins (123). Lowering the level of regulatory unfolded proteins results in ER stress which is linked to the unfolded protein response (124). Prolonged endoplasmic reticulum (ER) stress stimulates protein kinase-like ER-resident kinase (PERK), activates transcription factor-6 (ATF-6) and inositol requiring 1 (IRE1) apoptotic signaling (125). This leads to overexpression of C/EBP homologous protein (CHOP) expression, a proapoptotic-transcription factor (induced in ER stress), and is activated by p38 MAPK (126, 127). CHOP expression is usually regulated at mRNA level via PERK/eIF-2α/ATF6 pathway, ATF4 and IRE1/XBP1 pathway (128). Thus, to investigate the effect of Withaferin A in inducing apoptosis through ER stress, Choi et al. (129) conducted mRNA expression study of ER-stress specific X-box binding protein (XBP1) using human renal carcinoma cell line (Caki). Real-time PCR analysis showed that splicing of XBP1 mRNA was initiated following treatment with WA which in turn inactivates CHOP. Eukaryotic initiation factor-2α (EIF-2α) phosphorylation is activated by PERK which takes place during ER stress that leads to translational initiation and protein synthesis (130). These results suggest key signaling events implicated in ER-stress mediated apoptosis using WA which may facilitate chemopreventive and chemotherapeutic strategies based on Withaferin A (129).

“Adaptogens” are considered as plant-derived extracts or compounds which help organisms to adapt and survive during stress response (131). Activation of the gene that regulates stress response, which in turn postpones aging or ameliorates aging, is an effective way of tackling aging-associated diseases (132). Panossian et al. (133) studied adaptogens effect in stress and age-related diseases by using dunal root extract of WS in human T98G neuroglia cell line using mRNA expression profiling. It was reported that WS along with other adaptogens plays a major role in altering homeostasis. Also, it was observed that WS extracts downregulated PDE9A gene at transcriptional level. This indicated their potential to modulate transcriptional regulation in preventing aging-related and stress-induced disorders (133).

As people get older, the consistency or stability of circadian clock gene expression patterns is understood to potentially change or become less resilient (134). The suprachiasmatic nucleus (SCN) serves as the central master pacemaker, orchestrating the regulation of peripheral clocks found in all body tissues (135). In aging or in age-related complexities circadian timekeeping systems decrease (136). Circadian clocks operate through intricate transcriptional translational feedback loops (137). Two supplementary loops which include RORs, NFIL3, DBP and REV-ERBs, collaborate to produce discernible rhythms within a period of 24 h (138). Kukkemane et al. (139) evaluated WS influence on rhythmic alteration and levels on a daily basis with regard to age. They examined the expression of a hydroalcoholic leaf extract of WS using male Wistar rats. Through qRT-PCR analysis, they observed a significant chrono modulatory effect of WS. This effect was evident in the restoration of rhythms and pulses of clock genes namely Sirtuin1 and NRF2 daily. It was concluded that WS may offer therapeutic benefits in mitigating age-related disruptions in the circadian clock. This potential benefit is proposed to operate through two key pathways: SIRT1 modulation and antioxidant effects (139).

**Figure 7:**
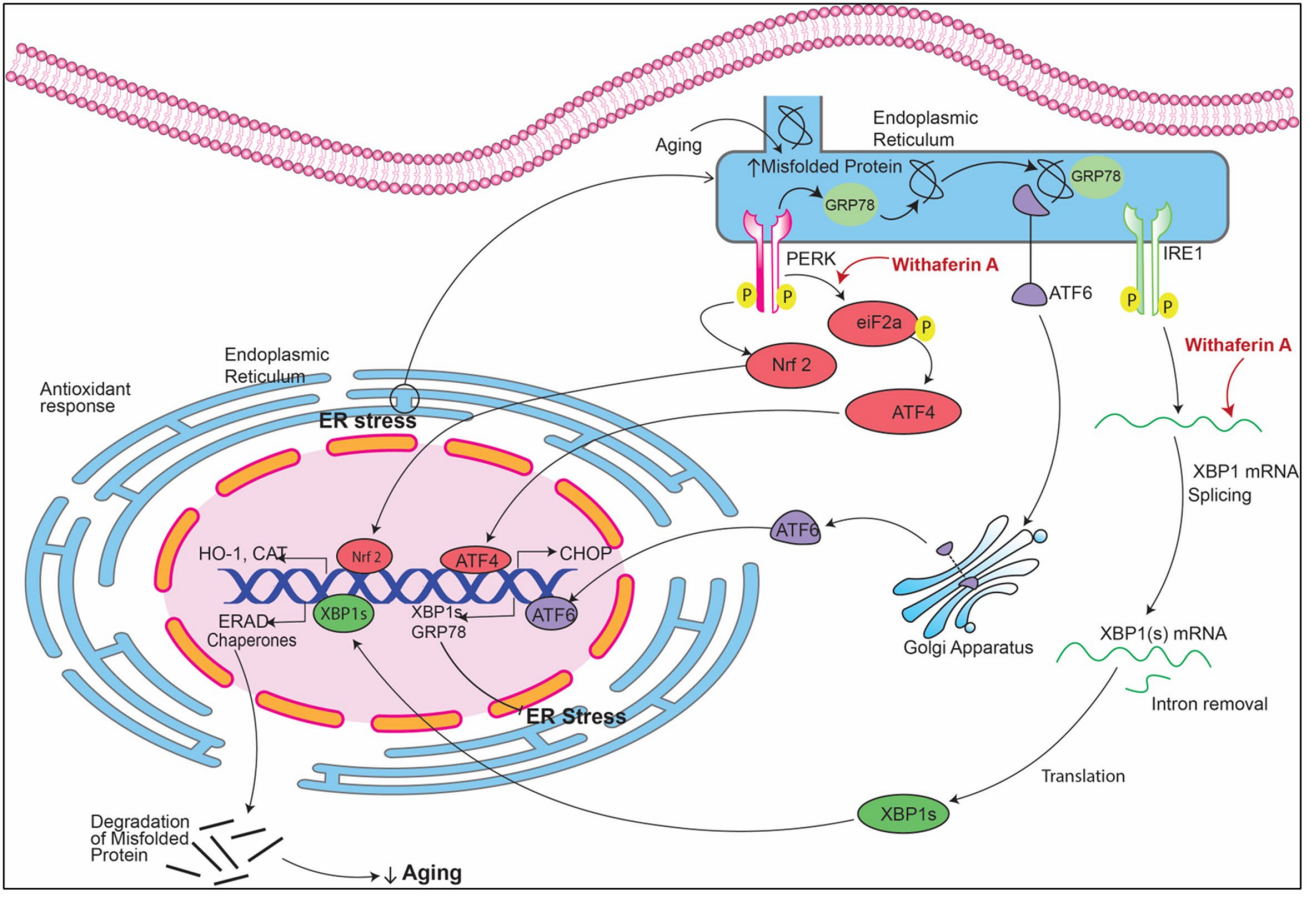
Signaling pathways implicating *Withania somnifera* in modulation of RNAs in ER stress. In ER stress, GRP78 (glucose-regulated protein) attaches to the misfolded proteins present in the endoplasmic reticulum (ER) which releases transmembrane proteins from endoplasmic reticulum (ER) namely inositol-requiring enzyme (IRE1), PRKR-like ER kinase (PERK) and activating transcription factor 6 (ATF6). These three proteins, when activated, initiate UPR signaling cascades. XBP1 mRNA is transcribed into XBP1s after cleaving by IRE1. PERK dimerization leads to phosphorylation of eIF2a that causes inhibition of protein translation. Withaferin A induces the splicing of XBP1 which in turn activates CHOP expression which resulting in inhibition of mitochondrial mediated apoptosis.

The regulation of signaling pathways in stress by *Withania somnifera* at RNA level are summarized in Figure 7.

#### 4.2.4 mRNAs involved in organ fibrosis

Fibrosis is characterized by endpoint of pathological remodeling which triggers the progression of various chronic disorders and aging-associated organ damage (140). Hence, it is important to alleviate the symptoms or inhibit the downstream pathways to prevent fibrosis. To investigate the potential role of Withaferin A as a preventive constituent in fibrosis, a study was conducted by Gu et al. (141) using male 8-week-old C57/BL6 mice. Silent information regulator (SIR) protein family exhibits an important role in cell cycle regulation, mitochondrial homeostasis maintenance, caloric restriction and lipid metabolism to regulate glucose levels. Studies have elucidated that SIRT3 deficiency can aggravate liver injury while SIRT3 activation led to alleviate liver fibrosis (142). In this study, mRNA expression level of SIRT3 was assessed after treatment of WA in C57/BL6 mice. It was reported that WA led to decrease of liver fibrosis in a SIRT3-dependent manner (141).

Aged kidney is susceptible to kidney injury which is indicated by extended inflammation that can increase dysfunction of renal tissue (143). NF-κB signaling pathway is a mediator between inflammation and fibrosis in kidney (144). Several stimuli can trigger NF-κB activity in the kidney such as TNF (145) and angiotensin II (146), and both of these are linked to chronic kidney disease. The target genes of NF-κB are long but include CCL2 and CCL5 which are linked to renal fibrosis (147). Therefore, to assess the role of WS in regulating these inflammatory genes, Grunz-Borgmann et al. (148) studied the expression using hot water soluble extracts of WS in rat kidney NRK-52E cell line. Transcriptomic analysis was performed using real-time PCR by using different doses of WS. It was reported that WS pre-treatment gradually inhibited CCL2 and CCL5 expression NRK-52E cells. Also, pre-treatment with WS reduced the effect of LPS-induced NF-κB activity indicating anti-inflammatory effect of WS and alleviating renal fibrosis (148).

Hepatic toxicity has been proved to be aggravated during aging especially by the consumption of acetaminophen (APAP) (149). APAP is generally used as an analgesic and antipyretic drug (150). However, when used in large concentrations, it causes hepatotoxicity and necrosis in humans (151). Devkar et al. (152) studied the outcome of withanolide-rich fraction (WRF) in liver protection using male Swiss albino mice. Expression of TNF-α, IL-1β, iNOS and COX-II was assessed at the transcriptional level in APAP-treated mice. It was reported that WRF significantly downregulated TNF-α and IL-1β transcriptionally corelating with the dose level. However, mRNA expression of iNOS and COX-II was reduced at higher dose (200 mg/kg) of WRF. Reduced expression of COX-2, iNOS, IL-1β, TNF-α achieved by WRF intervention showed anti-inflammatory and antioxidant properties of WS leading to enhanced liver protection (152).

**Figure 8:**
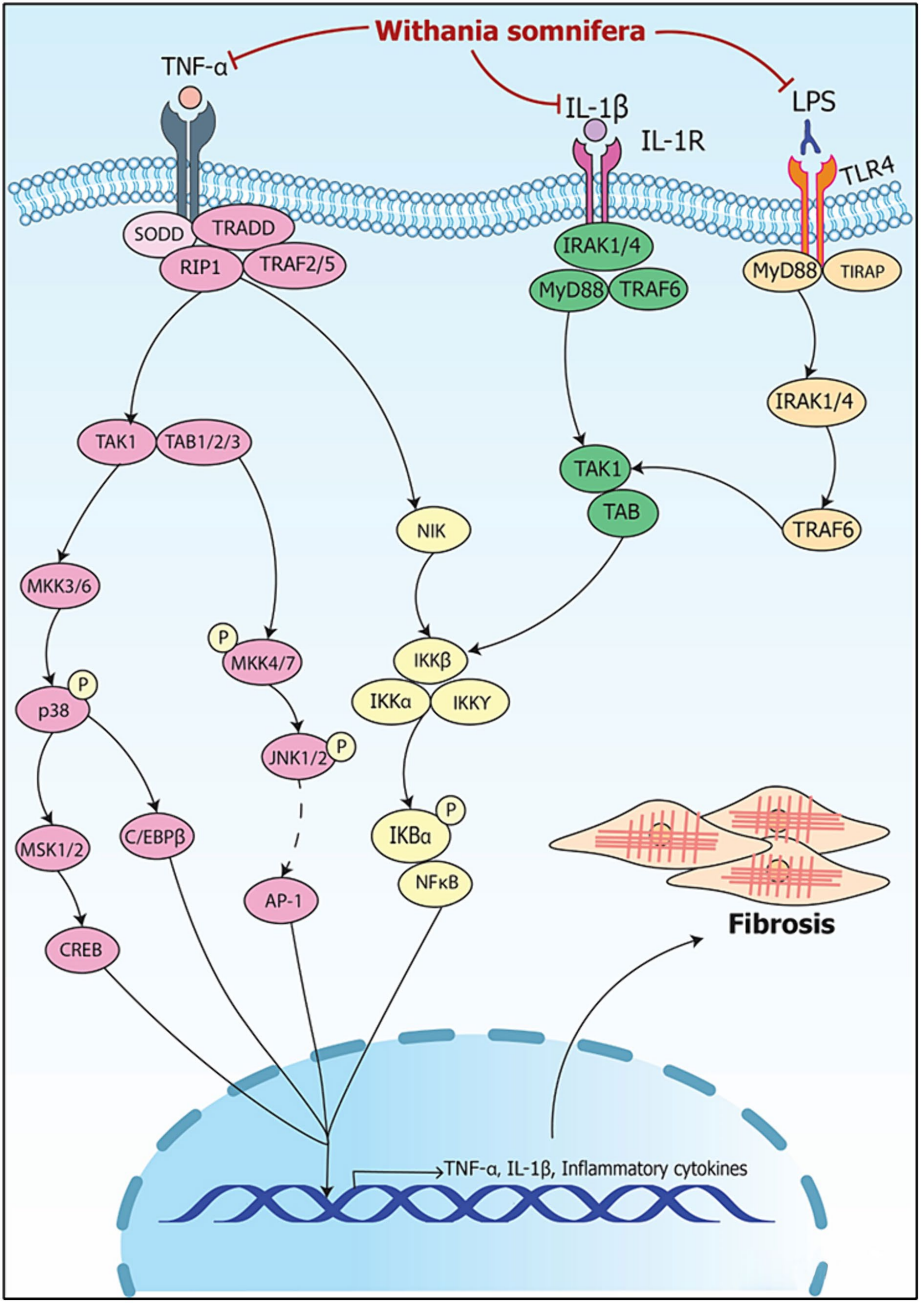
Signaling pathways implicating *Withania somnifera* in modulation of RNAs in organ fibrosis. TNF-α binds to TNFR and releases SODD leading to activation of TRADD/RIP1/TRAF2/5 complex; the complex activates TAK1/TAB1/2/3 and NIK; TAK1/TAB1/2/3 activates CREB and C/EBPβ via p38 and MKK4/7-JNK signaling. NIK activates IKK/NFκB signaling; IL-1 ligand receptor complex activates IRAK1/4/MyD88/TRAF6 complex further activating IKK/NFκB signaling. Moreover, LPS binds to TLR4 activating NFκB via TRAF6 signaling. CREB, C/EBPβ and NFκB results in the transcription of IL-1β and TNF-α. WS inhibits the binding of TNF-α, IL-1β and LPS to TNFR, IL-1R and TLR4, respectively, and suppresses inflammation resulting in attenuation of fibrosis.

The modulation of RNA in signaling involved in organ fibrosis by *Withania somnifera* is summarized in Figure 8.

#### 4.2.5 mRNAs involved in muscle impairment

The decline in mass and function of skeletal muscle typically commences around the age of 40, with muscle mass potentially diminishing at a rate of 1–2% annually after reaching 50 years of age (153). This can lead to escalating complications in health-related outcomes which lead to functional restriction of the body (154). During aging, reduced muscle building capacity leads to loss of muscle mass (154). It is believed that the loss of muscle due to aging is influenced by apoptosis, however the mechanisms behind it remain unclear (153). The Bcl-2 protein family is implicated in apoptotic signaling pathway, consisting of Bax and Bcl-2 proteins, which regulate the process of mitochondria-mediated cell death (155). While Bcl-2 helps prevent cell apoptosis, Bax, a protein like Bcl-2, promotes cell death (156). To understand the effect of WS on the regulation of apoptotic proteins, Panda et al. (153) conducted transcriptomic analysis using hydroalcoholic extracts of WS in male Sprague Dawley rats. Their study revealed that expression of Bax was reduced after the treatment with WS, along with an increase in Bcl-2 expression. It was therefore inferred that WS may offer a therapeutic approach to address muscle weakness and deterioration (153).

**Figure 9:**
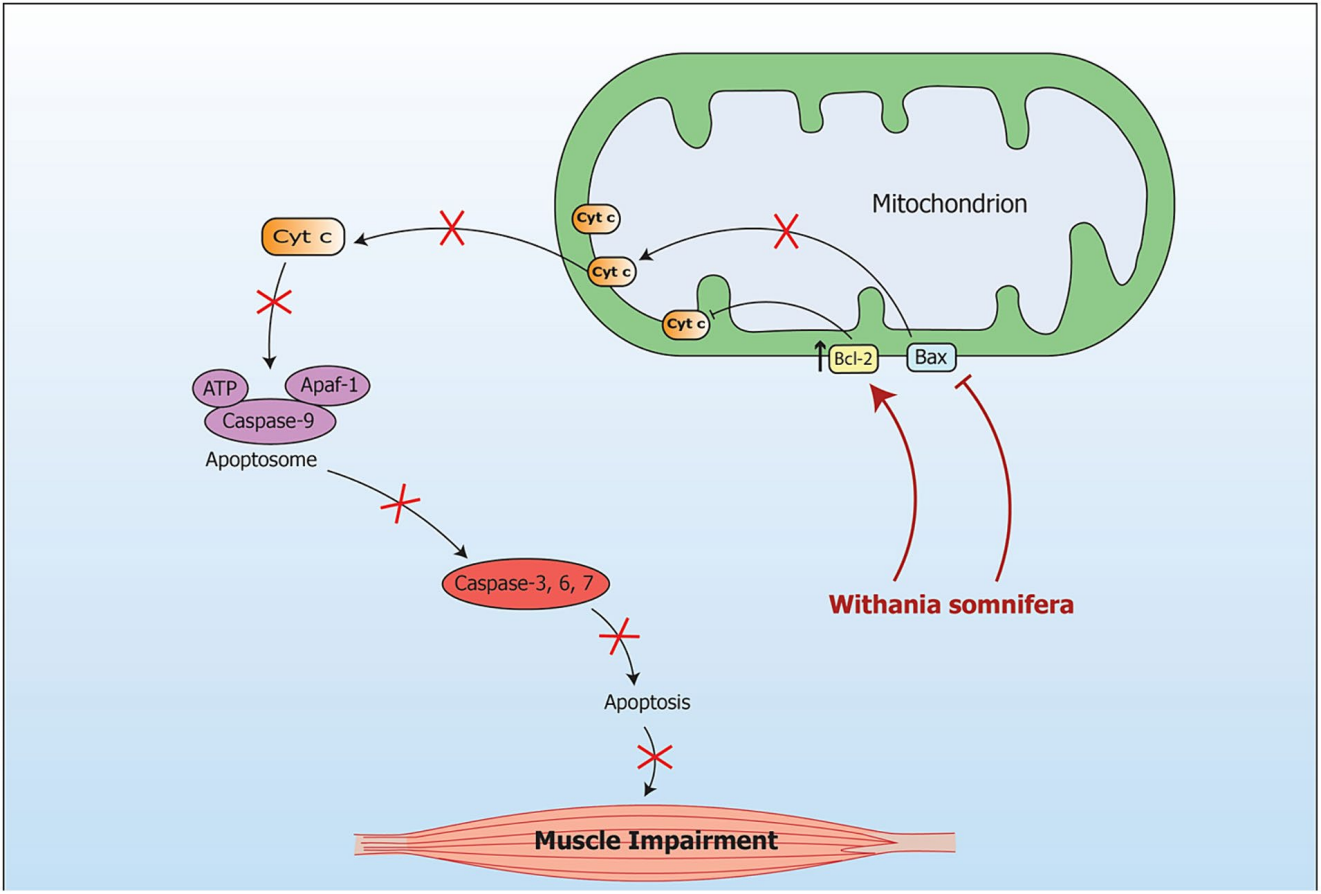
Signaling pathway implicating *Withania somnifera* in modulation of RNAs in muscle impairment. During oxidative stress, the mRNA level of Bax is increased while the mRNA level of Bcl is decreased in mitochondria. This leads to muscular apoptosis. Another pathway which is caspase dependent includes apoptosis due to stimulation of Caspase-3. Further, cytochrome c oozes out of the mitochondria in response to changes in Bax/Bcl-2 ratio. Introduction of WS inhibits Bax expression at transcriptional level which further inhibits apoptosis in muscle.

Signaling pathway modulated by *Withania somnifera* in muscle impairment is depicted in Figure 9.

#### 4.2.6 Miscellaneous mRNAs in aging

Telomerase shortening is considered as the main factor which escalates the rate of aging and promotes degeneration processes (157). Telomere is progressively shortened with each DNA replication, which leads to appearance of critically short telomeres (158). Low telomerase activity is targeted by both exogenous and endogenous factors which lead to DNA damage and low maintenance of cell function (159). Withanolide, an active constituent of WS indicated 29.7% extension in the lifespan of *C.elegans* via regulation of insulin/IGF-1 signaling pathway (160, 161).

Cellular senescence refers to cell-cycle suspension and secretion of inflammatory mediators which leads to aging and complications associated with aging (162). Obesity is an important comorbidity with increase in burden of senescent cell as well as neuropsychiatric disorders such as anxiety and depression (163). Withaferin A aids in alleviating several metabolic disorders. Therefore, to evaluate the protective effect of Withaferin A in conditions such as obesity, Abu Bakar et al. (164) performed a study using HFD-induced obese mice. They treated the mice with Withaferin A for 12 weeks. Pro-inflammatory cytokines were reduced following WA treatment. Additionally, using transcriptional analysis by real-time PCR, expression of all genes involved in inflammation such as TLR4, CCR2, NF-κB, COX2, TNF-α, IL-1β and CCL2/3 were downregulated following WA treatment. Also, WA downregulated the mRNA expression of main regulators of lipid metabolism (PPARα and γ, CD36, FAS and CPT1). These results suggested that WA treatment can help in regulating lipid and glucose metabolism in liver at the transcriptional level and provide a therapeutic response for WA in obesity (164).

Changes in vascular extracellular matrix in aging appear as one the major facets of dysregulated angiogenesis (165). In the process of angiogenesis, endothelial cells lead to the formation of new vasculature (166). Additionally, it is implicated in the progression of tumor cells and metastasis (167). The uniform release of VEGF, bFGF, PDGF and TGF lead to the activation of angiogenesis (168). Transcriptional as well as post-transcriptional mechanisms tightly regulate the expression level of VEGF mRNA (169). Recent research has unveiled the role of signaling pathways and genetic components governing this expression. The activation of the transcription factor Sp1 precedes VEGF promoter activity, showing that VEGF overexpression is regulated by Sp1 activation (170). This overexpression leads to angiogenesis and cancer progression (171). Sp1 has been evaluated as a crucial factor in tumor angiogenesis and aids in the pathogenesis of pancreatic adenocarcinoma (172). Santhekadur et al. (170) performed an experiment to explore the antiangiogenic effects elicited by WS, which resulted in a reduction in ascites fluid and VEGF expression, regulated by the Sp1 transcription factor, using Ehrlich ascites tumor (EAT) cells-bearing mice. Nuclear extracts were obtained from both untreated and withaferin A-treated EAT cells, and the binding activity of Sp1-DNA was studied using Electrophoretic Mobility Shift Assay (EMSA) with Sp1 oligonucleotides. The findings showed that Sp1 binding to VEGF gene promoter region was inhibited by withaferin A (170). Further, a study was carried out by Sajida and Prabhu (173) to analyze the activity of root extracts of WS using A549 human lung carcinoma cell line via inhibition mechanisms including antioxidant, autophagy and angiogenic inhibition. The expressions of VEGF, angiogenin and MMP-2 were assessed at the transcriptional level using qRT-PCR. It was reported that angiotensin and VEGF gene expression was significantly downregulated in WS extract-treated cells but there was a lower effect on MMP-2 expression (173).

**Figure 10:**
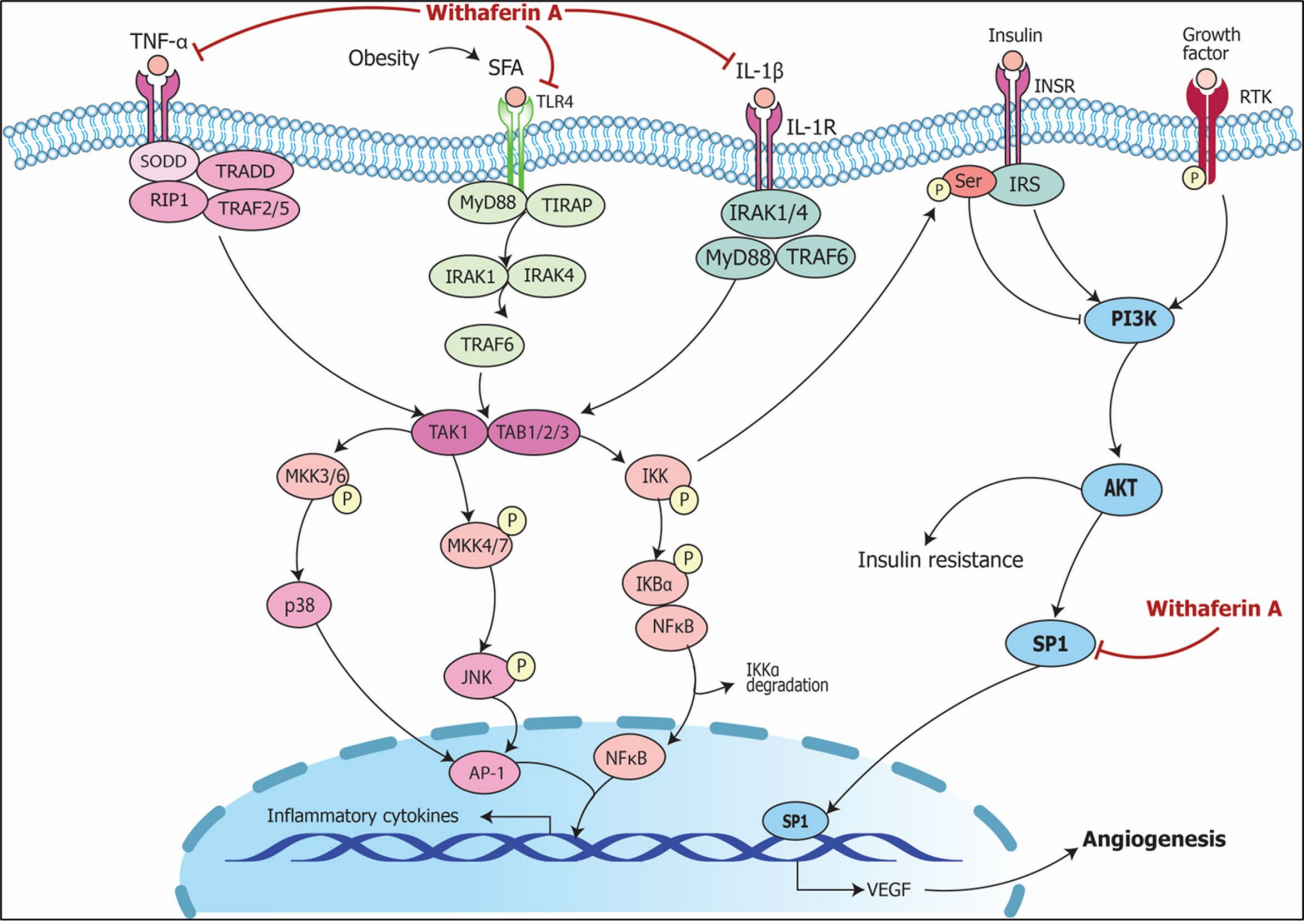
Signaling pathways implicating *Withania somnifera* in modulation of RNAs in other complications. Growth factor (GF) and receptor tyrosine kinase (RTK) binding leads to its phosphorylation which further initiates PI3K/AKT/SP1 signaling; RAS/RAF activates SP1 via MAP3K; both the events lead to transcription of VEGF resulting in angiogenesis. Withaferin A inhibits SP1 suppressing VEGF expression which results in the prevention of angiogenesis and oncogenic potential.

**Figure 11:**
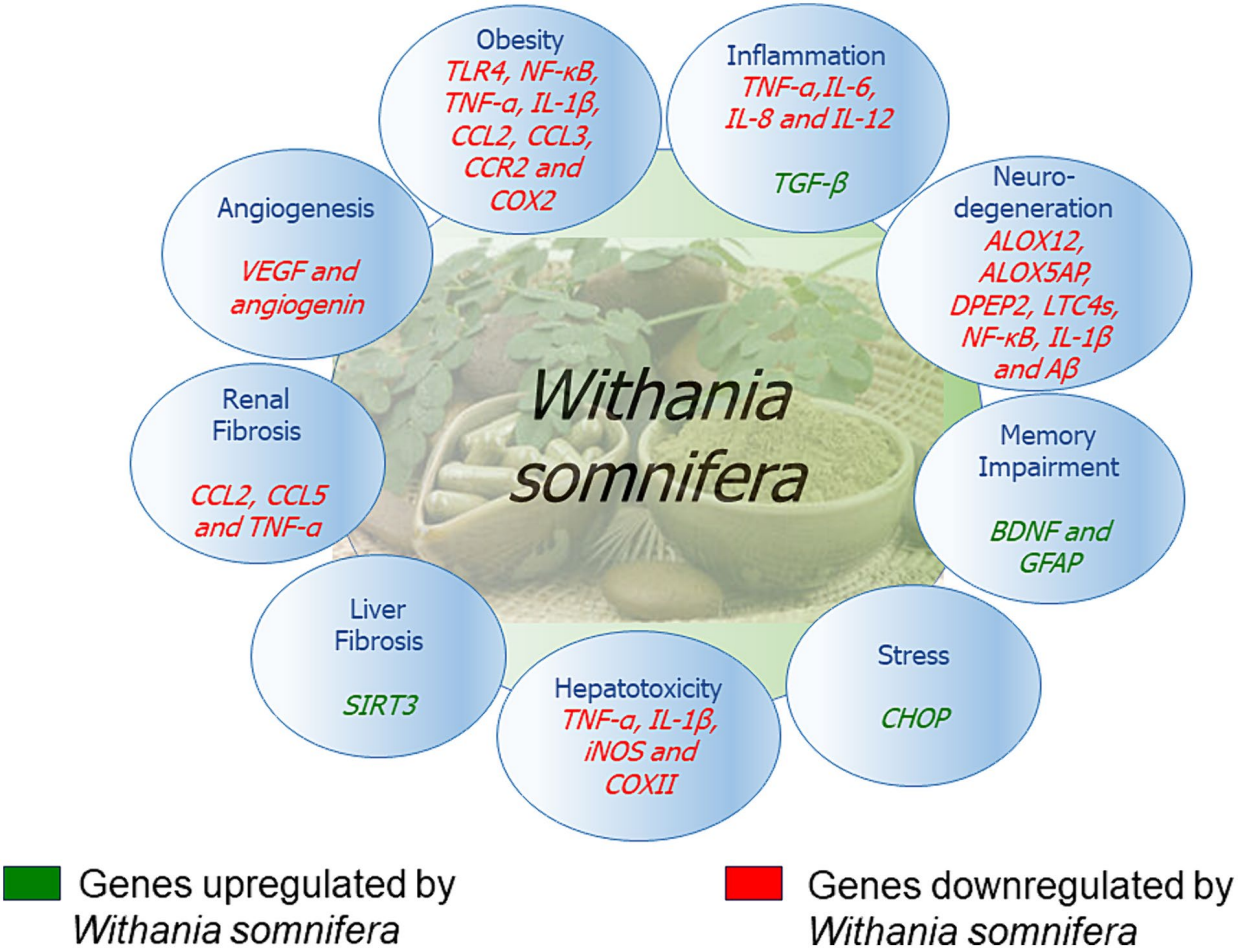
Genes modulated by *Withania somnifera*.

The signaling pathways involving *Withania somnifera* in modulating RNAs in above-mentioned cellular events are illustrated in Figure 10. In addition, RNAs modulated by *Withania somnifera* are represented in Table 1 and summarized in Figure 11.

**Table 1:** Modulation of RNAs by *Withania somnifera* in aging.

Sr.no	Disease phenotype	Biological matrix	RNA	Modulation	*Withania somnifera* source	Doses used	Toxicity or adverse events	Reference
1.	Stress and aging-related disorders	T98G neuroglia cells	PDE9A, PDE9S	*Withania* extract downregulated PDE9A gene	WS root extract 5 mg/mL (KSM-66)	5 mg/L and 1.5 mg/L	–	(133)
2.	Aging-related disorders which include inflammatory conditions, Alzheimer’s disease	T98G neuroglia cells	ALOX12, ALOX5AP, PTGER3, LTC4S, DPEP2, PLA2G16, PLA2G5, PLB1, PTGIS, PTGS2, PTGER2, PLA2G7, PTGFR, TBXA2R	*WS* downregulated ALOX12, LTCS4, DPEP2 and ALOX5AP expression	WS roots, withanolide 5.5%	–	–	(174)
3.	Obesity	Male C57BL/6 J mice	mRNA expression of PPARγ, CD36, FAS, LXRα, SREB P-1c, PPARα, CPT1, and ACC	WA downregulated the expression of PPARα, CS36, CPT1, PPARγ and FAS at transcriptional level	Withaferin A (WA) purity ≥95%	1.25 mg/kg/day	–	(164)
4.	Liver Fibrosis	C57/BL6 mice	mRNA expression of SIRT1 to SIRT7	WA inhibited SIRT3	Withaferin A of purity ≥99%	CCl4 + WA 2.5 mg/kg and 10 mg/kg	–	(141)
5.	Psoriasis, allergic contact dermatitis and atopic dermatitis	Human keratinocyte cell line HaCaT	IL-6, IL-8, IL-12 TGF-β and TNF-α mRNA	ASH-WEX inhibited expression of IL-6, IL-8, IL-12 and TNF-α. ASH-WEX increases TGF-β expression	Water extract of Ashwagandha roots	0.25 and 1.5 mg/mL	–	(112)
		7-week-old male C57BL/6 J mice				10 mg/mL		
6.	Anticancer effect through ER stress	Renal carcinoma cell line (Caki)	X-box binding protein (XBP1)	WA induces XBP1 splicing which results in CHOP inactivation.	Withaferin A purchased from Biomol	2, 4, 6 μM for 5 h	–	(129)
7.	Hepatic toxicity	Male Swiss albino mice	TNF-α, COX-II, iNOS and IL-1β	WRF decreased *TNF-α* and *IL-1β* expression	Withaferin-rich fraction isolated from *Withania somnifera* roots	50, 100 and 200 mg/kg	–	(152)
8.	Osteoarthritis	Rabbit articular chondrocytes	COX-II	WA increased the expression of miR-25 by inducing COX-2	Withaferin A (Calbiochem)	3 μM WA	–	(105)
9.	Alzheimer’s Disease	SH-APP cells	NF-κB, IL-1β, IL-6, Aβ	WA downregulated NF-κB, IL-1β and Aβ expression WA upregulated IL-6 expression	Withaferin A (Sigma Aldrich)	1 μM WA	–	(119)
10.	Renal Fibrosis	Rat kidney NRK-52E cell line	NF-κB, CCL2, CCL5	*Withania somnifera* downregulated the expression of NF-κB, CCL2 and CCL5	Water soluble extracts of Ashwagandha (Now Foods, Bloomingdale, IL, USA)	–	–	(148)
11.	Memory impairment	Swiss male albino mice	BDNF and GFAP	*Withania somnifera* downregulates the effect of scopolamine hence increasing BDNF and GFAP expression	Alcoholic extract of WS leaves	100, 200 and 300 mg per kg	–	(122)
12.	Lung adenocarcinoma	A549 human lung adenocarcinoma cell line	VEGF, angiogenin and MMP-2	*Withania somnifera* significantly downregulates the expression of VEGF, angiogenin and had lower effect on MMP-2	Ethanolic extracts of *Withania somnifera*	12.5, 25, 50, 100 and 200 μg/mL	–	(173)
13.	Circadian rhythms	Male Swiss Wistar rats	SIRT1, NRF2 and *rRev-erba*	*Withania somnifera* restored the expression of SIRT1 and NRF2	Hydro-alcoholic leaf extract of *Withania somnifera*	–	–	(139)
14.	Muscle strength	Male Sprague Dawley rats	Bax and Bcl-2	*Withania somnifera* significantly decreases Bax expression and significantly increases Bcl-2 expression	Hydro-alcoholic extract of Ashwagandha	500 mg/kg	–	(153)
15.	Telomere strength	*C. elegans*	IIS (insulin/IGF-1) cascade	WA upregulated *skn-1* and *gst-4 (a major components of IIS)*	Withanolide A (Natural Remedies)	5 μM	–	(161)
16.	CVD	Human umbilical vein endothelial cells (HUVECs).	lncRNA H19	*Withania somnifera* increases the effect of H19	–	–	–	(175)
17.	Anti-angiogenic effect	Ehrlich ascites tumor (EAT) cells bearing mice	Sp-1	*Withania somnifera* inhibits the effect of Sp-1	Withaferin A	7 mg/kg/day/mouse	–	(170)
18.	Anxiety and neuroinflammation	Wistar albino mice	PPARγ, iNOS, MCP-1, TNF-α, IL-1β, IL-6, Bax and Bcl-2	WS downregulated MCP-1, IL-6, TNF-α, IL-1β, iNOS, PPARγ, Bax and Bcl-2	Leaf powder extract of WS	–	–	(113)

## 5 Sequencing studies with *Withania somnifera* in aging/inflammation

Panossian et al. (133) studied the expression of various plant-based adaptogens using mRNA sequencing to infer the role of plant extracts in alleviating complications that are concomitant with aging and stress. They performed RNA-seq to assess changes in gene expressions in adaptogen-treated T98G human neuroglia cells. The functional relevance of dysregulated genes to adaptive stress signaling was analyzed. Seventy-five genes were found to encode various biological components, which includes enzymes used in metabolism; neurohormones such as CRH, UCN, GNRH1; GPCR and transmembrane receptors like PLXNA4, GPR19, TLR9, PRLR, GPR158, GP1BA, VIPR2, CHRNE; transmembrane channels; protein kinases including FLT1, ROS1, MAPK10, MERTK, TTN, MAPK13, PRKCH; ligand-dependent nuclear receptor RORA; phosphatases namely PTPRD, PTPRR; transcription regulators such as ZNF467, FOS, SCX, ZFPM2, ZNF396, STAT5A, FOXO6; peptidases such as TLL1, PAPPA2; and various other proteins (133). These findings indicated that adaptogens might impact multiple adaptive stress-response signals, including those related to cyclic AMP, MAPK, NRF2 oxidative stress, glucocorticoid receptor (GR or NR3C1), maturation of neurons as well as CREB pathway which occurs in neurons and synapses, corticotropin releasing hormone, AMPK, melatonin, endothelial nitric oxide synthase, nutrient sensing in enteroendocrine cells mediated by GPCR, Gαs, GP6, opioid, renin-angiotensin and conditions such as neuropathic pain, and neuroinflammation. These results suggested that adaptogens provide a diverse biological function, eliciting numerous effects in regulating cellular metabolism and maintaining homeostasis at the transcriptional level (133).

Moreover, another study by Panossian et al. (174) elucidated anti-inflammatory properties of WS using T98G human neuroglia cell line using mRNA sequencing. A total of 14 genes which were included in eicosanoids pathway were altered by WS extracts. This includes arachidonate 5-lipoxygenase activating protein (ALOX5AP), GPCR such as PTGFR, TBXA2R, PTGER2, PTGER3, and several enzymes. It was inferred that downregulation of ALOX12 expression plays a key role in neurotoxicity. In addition, WS downregulated ALOX5AP, leukotriene C4 synthase (LTC4S) and DPEP2 genes involved in biosynthesis of leukotrienes pathway resulting in its inhibition. This, in turn, inhibited neuroinflammation and neurodegeneration leading to the suppression of Alzheimer’s disease complications. Also, PTGER3 expression was increased by WS which aids in ulcer prevention in the duodenum and small intestine. Moreover, EP3 receptor genes are upregulated in breast cancer patients. Therefore, suppression of PTGR3 can be used in treating breast cancer. Combination of *Rhodiola* and *Withania* inhibited LTC4 signaling by downregulation of LTC4S and upregulation of PTGER3 at genetic level which may serve as a promising approach in treating allergic asthma (174).

## 6 Clinical trials including RNA markers in aging and inflammation

Several trials have been performed in various parts of the world to study the effects of RNA markers (which include mRNAs and non-coding RNAs) on various aging and inflammation-related conditions such as CVD, neuroinflammatory and neurodegenerative complications, as well as complications related to metabolism in humans. Clinical trials data have been extracted from ClinicalTrials.gov (176), EU Clinical Trials Register (177) and Brazilian Registry of Clinical Trials (ReBEC) (178). These trials aim to evaluate the RNA-based interventions, as well as to identify biomarkers of response and resistance. These results may provide new understanding about aging and inflammation and insights into their mechanisms at the molecular level. This will pave the way for the advancement of novel therapeutic and preventive strategies in aging-related diseases. Clinical trials including RNA markers in aging and inflammation are summarized in Tables 2, 3.

**Table 2:** Clinical trials including RNA studies in aging and inflammation.

Registry ID	Trial status	Trial phase	Participants (*n*)	Medical complications	Objective of the trial	Dose used	References
NCT02432287	Completed	Phase 4	16	Aging	To assess gene expression pattern in older individuals with glucose intolerance back to a profile similar to that of young, healthy subjects	1700 mg/day	(179)
NCT01480037	Completed	–	38	Aging; without mention of psychosis	To determine the gene expression in association with oxidative stress in the peripheral blood of the three observed groups, we’ll be utilizing the Superarray—RT2 Profiler™ PCR Array System	–	(180)
NCT03300388	Completed	–	85	Obesity, Aging and Inflammation	To analyze the RNA and miRNA expression in adipose tissue	1.650 mg/day of DHA	(181)
NCT05235958	Enrolling	–	43	Cardiovascular risk, sedentary lifestyle and endothelial function	To measure various micro-RNAs that play pivotal roles in regulating endothelial function within the macrovascular circulation	–	(182)
NCT03104075	Completed	Phase 4	40	Pneumonia and aging	To assess quantitative analysis using RNA-seq and ATAC-seq methods to elucidate the epigenetic makeup of immune cells concerning their reaction to the vaccine	Prevnar 13 and Pneumovax 23 (Pneumococcal vaccine) 0.5 mL dose	(183)
NCT03056105	Completed	–	66	Aging	To assess expression of telomere regulated- and inflammation-related genes	–	(184)
NCT03289832	Completed	–	25	Healthy adults	To assess modulation in tissue-based RNA biomarkers of inflammation and alteration of aging-related RNA markers	Crucera-SGS^®^ (450 mg/day) and Meriva 500-SF^®^ (1,000 mg/day)	(185)
NCT01508091	Unknown	–	48	Obesity	To measure UCP3 mRNA expression in vastus lateralis	–	(186)
NCT05062707	Recruiting	–	120	Cancer	To assess early aging- and senescence- related markers	–	(187)
NCT03464500	Completed	–	90	Muscle function, mitochondrial function, overweight, healthy aging	To assess change in the gut microbial diversity via 16 s RNA sequencing performed on faecal samples.	Mitopure 500 mg and Mitopure 1,000 mg	(188)
NCT02132091	Completed	–	37	Aging, metabolism	To assess effect of intermittent fasting on aging- and oxidative stress- related markers	One gram of Vit. C and 400 IU of Vit. E	(189)
NCT04079218	Active, not recruiting	Phase 4	51	Aging, HIV infection, Vaginal atrophy, Menopause, Premature aging, Dysbiosis	To determine the effect of HIV on genital tract of aged women and to study the effect of drug efficacy in postmenopausal women suffering from HIV	Estradiol tablet (10 μg), intravaginally everyday (2 weeks)	(190)
NCT05895591	Completed	–	33	Anti-aging	To assess clinical efficacy of anti-aging cream.	–	(191)
NCT04199195	Enrolling by invitation	–	360	Aging	To study the function of gut microbiome during aging to provide insights for improving the lifestyle of elderly	–	(192)
NCT05593939	Completed	Phase 2	80	Aging	To assess and measure the slow aging inn humans by using various interventions	Nicotinamide riboside tablets 1 g in morning and 1 g in evening	(193)
NCT04945265	Not yet recruiting	–	500	Female infertility	To provide insights into granulosa cell function which is the root cause of fertility	–	(194)
NCT06096532	Not yet recruiting	–	24	Healthy, Aging	To investigate the adipose tissue flow in elderly	Oral glucose 75 gm	(195)
NCT02953093	Completed	Phase 2	10	Aging	To assess changes in gene transcription of fat and muscle tissue with acarbose treatment	Acarbose	(196)
NCT04156048	Completed	–	110	Intrinsic Aging of Skin	To discover function of retinol on skin in aged individuals	Retinol	(197)
NCT01333644	Completed	–	270	HIV infection, Cardiovascular disease, Inflammation	To study the role of inflammation and aging in HIV-associated cardiovascular complications	–	(198)
NCT05190432	Active, not recruiting	–	90	Antioxidative stress, Cold, Influenza, Aging, Inflammation	To study the effect of Taxifolin and Ergothioneine on immune systems in healthy population	Taxifolin 250 mg/day and Ergothioneine 80 mg/day	(199)
NCT05053282	Unknown status	–	60	Aging	To assess the role of endurance exercise on regulation of circadian rhythm and physiology in aged individuals	–	(200)
NCT06091969	Not yet recruiting	Phase 2	64	Male fertility	To study nutraceutical supplementation for male subfertility	Fertility enhancer, FE daily for 3 months	(201)
NCT05008770	Recruiting	–	110	Sarcopenia	To evaluate musculoskeletal complications in aged individuals as a result of sarcopenia	–	(202)
NCT05798637	Recruiting	–	200	Coronary artery disease (CAD), T2DM and AD	To evaluate frailty associated inflammation in aged individuals and to study the role of blood platelets	–	(203)
NCT06082362	Recruiting	–	350	Male Infertility	To personalized key markers of chronic inflammation and early aging in infertile men	–	(204)
NCT04691986	Recruiting	–	144	Sarcopenia, NAD contraception, Muscle quantity and NAD+ content	To study the aftermath of NR (nictotinamide roboside) on functional ability and physiology of muscle tissue in elderly	NR supplementation at 1,000 mg/day	(205)

Adapted from www.clinicaltrials.gov (176).

**Table 3:** Clinical trials including RNA studies in aging and inflammation.

Clinical Trial Registry	Registry ID	Trial status	Trial phase	Participants (*n*)	Medical complications	Objective of the trial	Dose used	References
Europe	2021–001654-65	Ongoing	Phase 2	–	Chronic obstructive pulmonary disease (COPD)	To study the of course of action of drug itepekimab on airway-related inflammation treated with COPD	–	(177)
Europe	2015–002682-30	Completed	Phase 4	–	Aging-related inflammation in HIV patients	To investigate the difference in IL-6 marker in patients treated with protease inhibitor and raltegravir which is used with or without the use of statins	–	(206)
Brazil	U1111-1218–7442	Recruitment completed	Phase 2	120	Gingivitis	To study the effectiveness of a long-term medication for gingival inflammation therapy	–	(207)

Adapted from European Clinical Trial Registry (177) and Brazilian Registry of Clinical Trials (178).

## 7 Conclusion and future perspectives

Management of aging is difficult due to its progressive and irreversible nature, as well as the comorbidities associated with aging. However, the quality of biological aging can be improvised by recent advancements including intervention with nutraceuticals that can modulate the transcriptional activity of different genes implicated in aging and age-related complications. We have discussed the role(s) of *Withania somnifera* in modulating RNAs and alleviating the aging-associated comorbidities. Interestingly, Martínez-Mármol et al. (208) revealed that in brain organoids, long COVID prompted fusion among neurons and between neurons and glial cells. This fusion was attributed to a viral fusogen induced by the infection, driven by S-protein expression of SARS-CoV-2. These findings reveal how the nervous system is impacted by COVID-19 virus, potentially causing various neuropathologies. This indicates a potentially beneficial role for WS in long COVID management. However, the specific involvement of WS in the development of neuropathologies resulting from SARS-CoV-2 infection remains to be fully understood.

With the advent of new high-throughput ‘omics’ technologies and updation of the reference human genome, we are now better placed to investigate aging at the transcriptional level. Indeed, the field of RNA biology has grown tremendously with the development of antisense oligonucleotides (209, 210), aptamers (211), RNA vaccines (212), siRNAs (213, 214) and CRISPR/Cas9 gene editing (215). Further, the platforms used for RNA delivery are also expanding with nanocarriers (216), lipid nanoparticles (217) and exosomes (218) being evaluated as novel drug delivery systems. Future avenues of research will likely include a deeper understanding of *Withania* effects on the transcriptome by the use of next-generation sequencing followed by formulation of *Withania* in a suitable dosage form to modulate the genome. In this review, we summarize the promising effects of *Withania* in ameliorating age-related molecular changes by encompassing both *in vitro* and *in vivo* studies. We have also summarized clinical trials studying modulation of RNAs in aging to provide current insights into possible interventions for aging-related complications. Moreover, we delineate key signaling pathways regulated by *Withania* compounds and key aging biomarkers in the aging/inflammation process. Taken together, given the modulation of key RNA markers in aging and inflammation pathways, there is tremendous potential for harnessing the beneficial effects of *Withania* for achieving healthy aging.

## Author contributions

PS: Writing – original draft. SA: Writing – original draft. DMa: Writing – original draft. SS: Writing – original draft. DMe: Writing – review & editing. SN: Supervision, Conceptualization, Project administration, Writing – review & editing.
